# Efficacy of an iron‐fortified infant cereal to reduce the risk of iron deficiency anemia in young children in East Cameroon

**DOI:** 10.1002/fsn3.1639

**Published:** 2020-06-04

**Authors:** Tetanye Ekoe, Ousmaila I. Bianpambe, Felicitee Nguefack, Daniel M. Pondi, Marie M. Kana‐Sop, Nicholas P. Hays, Gabriel Medoua, Paul N. Koki

**Affiliations:** ^1^ Faculty of Medicine and Biomedical Sciences University of Yaoundé I Yaoundé Cameroon; ^2^ Faculty of Sciences University of Douala Douala Cameroon; ^3^ Nestlé Product Technology Center – Nutrition Vevey Switzerland; ^4^ Food and Nutrition Research Center Institute for Medical Research and Study of Medicinal Plants Yaoundé Cameroon

**Keywords:** Africa, anemia, cluster‐randomized controlled trial, iron fortification, nutritional status, under‐five children

## Abstract

Complementary foods in Africa are often poor sources of bioavailable iron. We assessed the efficacy of iron‐fortified wheat‐based infant cereal (IC) to reduce the risk of iron deficiency anemia in children aged 18–59 months in Cameroon. A 6‐month double‐blind, cluster‐randomized controlled trial was conducted in 2017 among anemic (hemoglobin 7–11 g/dl) but otherwise healthy children. In conjunction with usual diet, children received two 50 g servings/day of a standard, micronutrient‐fortified IC (providing 3.75 mg iron/serving; *n* = 106) or the same IC without iron fortification (*n* = 99). Anthropometric measurements, blood sampling, and systematic deworming were performed in all children at baseline (pre‐intervention), 3, and 6 months. Mean hemoglobin, ferritin adjusted for C‐reactive protein (CRP), serum iron, transferrin saturation, prevalence of anemia, iron deficiency, and iron deficiency anemia as well as anthropometrics were compared between the groups at baseline, 3, and 6 months. Compared to the control group, children consuming the iron‐fortified IC had significantly higher baseline‐adjusted mean hemoglobin (10.0 ± 1.8 vs. 9.7 ± 1.4 g/dl, respectively; *p* = .023), ferritin adjusted for CRP (16.1 ± 8.3 vs. 9.5 ± 7.5 μg/L, *p* < .001), serum iron (14.5 ± 3.9 vs. 11.2 ± 4.4 μg/dl; *p* < .001), and transferrin saturation (19.0 ± 17.4 vs. 10.7 ± 12.5%; *p* ˂ .001) at 6 months. The prevalence of anemia, iron deficiency, and iron deficiency anemia at 6 months decreased by a larger extent in the iron‐fortified group versus controls (all *p* < .01). In addition, at 6 months, children in the iron‐fortified group demonstrated higher weight‐for‐age *z*‐scores (*p* = .016) compared to the control group. Wheat‐based IC fortified with 7.5 mg ferrous fumarate administered daily for 6 months improved iron and nutritional status and decreased the prevalence of iron deficiency anemia in children aged 18–59 months in Salapoumbé, Cameroon.

## Graphical abstract

Consumption of micronutrient‐fortified wheat‐based infant cereal providing 7.5 mg/day of iron fumarate for 6 months improved iron status and decreased the prevalence of iron deficiency anemia in children aged 18–59 months.

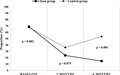

## 1 INTRODUCTION

Anemia and iron deficiency in infants and children can negatively affect growth, cognitive development, academic performance, and immunity (Beard, 2008; Dallman, Siimes, & Stekel, 1980). Anemia affects nearly half of all children under 5 years of age, or about 273 million children, with the highest prevalence found in developing countries (McLean, Cogswell, Egli, Wojdyla, & de Benoist, 2009). In Cameroon, 60% of children under 5 years of age are anemic, mainly due to iron deficiency (Institut National de la Statistique (INS) and ICF International, 2012) although other factors such as malaria, helminth infections, and deficiencies of vitamin B12, vitamin A, or folate may also contribute (Engle‐Stone, Aaron, et al., 2017; Loukas et al., 2016). It has been established that iron and other micronutrient deficiencies can be prevented by dietary diversification (i.e., encouraging the consumption of micronutrient‐rich foods that may be available but underutilized by the population), supplementation, and fortification of widely consumed foods (Das, Salam, Kumar, & Bhutta, 2013; Eichler, Wieser, Ruthemann, & Brugger, 2012; World Health Organization & Food and Agricultural Organization of the United Nations, 1992). The World Health Organization (WHO) has reported that iron fortification of foods is the best preventive measure of iron deficiency from a cost‐effectiveness point of view (Baltussen, Knai, & Sharan, 2004; Horton, 2006). Although several studies have confirmed the efficacy of iron‐fortified foods on improving iron status (Bouhouch et al., 2016) and correcting iron deficiency anemia (Andang'o et al., 2007; Sun et al., 2007) in South America, Asia, and Africa, other studies reported mixed or negative results (Assuncao, Santos, Barros, Gigante, & Victora, 2007; Glinz et al., 2015, 2017; Nestel et al., 2004; Rohner et al., 2010). A systematic review of both efficacy and effectiveness trials concluded that consumption of iron‐fortified foods results in improved iron status and reduced risk of anemia, yet significant heterogeneity was observed for most outcomes (Gera, Sachdev, & Boy, 2012). Another systematic review of flour fortification programs in 13 countries (none located in Africa) concluded that only limited evidence exists for the effectiveness of iron‐fortified flour in reducing anemia prevalence (Pachon, Spohrer, Mei, & Serdula, 2015), perhaps due to variability in program implementation (e.g., compliance, coverage, feasibility in rural settings), iron compound, and iron levels. Together, these results suggest the need for further studies on the efficacy of iron fortification.

Per WHO recommendations, several countries have routinely adopted the strategy of using iron‐fortified foods to combat iron deficiency (Nutrition International, 2018). In Africa, 26 of 53 countries including Cameroon have officially adopted iron fortification of cereal flours to reduce the burden of iron deficiency anemia in children (Food Fortification Initiative, 2018). In Cameroon, iron is added to wheat flour as ferrous fumarate (60 mg Fe/kg) (Food Fortification Initiative, 2018). The time and effort to prepare complementary foods from fortified flour rather than from the same foods eaten by older family members, however, may prevent the successful implementation of this policy in all households. For example, in rural Cameroon, complementary foods are often traditional meals (e.g., maize paste and okra sauce) and made with family staples (potatoes, cassava, rice, beans, and peanuts), with infrequent usage of wheat flour (Mananga, Kana‐Sop, Nolla, Tetanye, & Gouado, 2014). Furthermore, the finding that only ~75% of wheat flour samples in Cameroon were actually fortified, plus the lack of improvement in hemoglobin concentration or anemia prevalence in response to wheat flour fortification in Cameroonian children (who commonly receive antihelminthic medications [Institut National de la Statistique (INS) and ICF International, 2012]), suggests that other interventions are needed (Engle‐Stone, Nankap, et al., 2017). Nutrient‐rich foods that are consistently fortified, convenient to prepare, and readily accepted by young children, such as commercial infant cereal, are important options that can help improve iron consumption during this crucial period of child development. Therefore, we conducted a cluster‐randomized controlled trial to evaluate iron fortification of commercial wheat‐based infant cereal (IC) to reduce the risk of iron deficiency anemia in young Cameroonian children.

## 2 METHODS

This double‐blind, cluster‐randomized, controlled trial was conducted from February to August 2017 in the locality of Salapoumbé (Cameroon). A random sample of 366 children from the entire locality was initially selected within 30 villages based on local vaccination registers. Further inclusion criteria for this study included age 18–59 months, hemoglobin level between 7 and 11 g/dl and apparent good health. Exclusion criteria were also applied, including iron supplementation in progress per caregiver report, clinical presentation of severe malnutrition (e.g., bilateral pitting edema), diagnosis of any chronic infection (e.g., tuberculosis, HIV), severe acute infection (e.g., severe malaria, pneumonia, meningitis), blood transfusion <3 months prior to enrollment, or allergy/intolerance to cows' milk and/or gluten. From the initial sample of 366 children, 205 were eligible after inclusion and exclusion criteria were applied. Children with weight‐for‐height *z*‐scores <−3 were not excluded; however, only six of the 205 children had scores <−3 at baseline.

The minimum sample size was calculated for 90% statistical power and α‐level of 5%, with a predicted difference for mean hemoglobin of ≥0.9 g/dl and a standard deviation 1.48 g/dl (Eichler et al., 2012) in the iron‐fortified group between baseline and study completion at 6 months. The minimum sample size was 71 children per group (142 subjects total). To account for dropouts, the entire eligible population of 205 children was enrolled.

The Salapoumbé locality is in the East region of Cameroon that is quite rural and known to be malaria endemic. Salapoumbé is divided by the Lokomo River, which is large and difficult to cross, thus separating the locality into two settlements. The two settlements are generally ethnically comparable in eating habits and customs as well as at the socioeconomic level, notably in terms of literacy and agriculture. Thus, the south and north banks were considered two clusters and the IC (fortified or control) was randomized by cluster. This design was implemented to minimize treatment crossover due to potential sharing of IC between families located on the same bank of the river.

The batches of IC were manufactured by Nestlé Central and West Africa Ltd. PMB KIA Accra‐Ghana according to international cereal fortification standards (Hurrell et al., 2010). Bags of IC were packaged in waterproof plastic and were identical in appearance except for a code (A or B) designating whether the batch was iron‐enriched. The code assignment was unknown to researchers and subjects throughout the study period. The random allocation of IC A or B to the south or north bank was performed by the principal investigator by a flip of a coin. All subjects on each bank received the same IC, either A or B. The study team obtained the support of administrative officials and traditional chiefs of the respective villages to ensure monitoring of the correct distribution of the IC lots. Community health workers also verified through periodic home visits that children enrolled in the study were receiving the IC packets corresponding to the randomized area. The manufacturer sent the batch code assignment to the principal investigator after the statistical analysis was complete.

Parents of enrolled children completed a sociodemographic characteristics questionnaire based on a protocol previously validated by the Comité National d'Ethique et de la Recherche pour la Santé Humaine du Cameroun (CNERSH). Data obtained included the vaccination history of the child, the occurrence of illness during the previous 2 weeks, and the child's date of birth. Child anthropometric assessments included weight measurement using an electronic portable scale (SECA LK 5051) with a sensitivity of 0.5 g. The scale was calibrated after every 10 measurements. Length or height was measured using a SECA portable infantometer or stadiometer with an accuracy of 0.1 cm. Immediately after the anthropometric measurements were taken by trained personnel, each child's hemoglobin level was measured with a portable hemoglobinometer (HemoCue 201+). A 3 ml sample of venous blood was collected and centrifuged, and the serum was immediately frozen for analysis by a Biotek EL800X machine to measure levels of ferritin, serum iron, transferrin, and C‐reactive protein (CRP). All assays were performed according to manufacturer's instructions. Appropriate procedures (e.g., portable freezers or generators) were used in the field to maintain blood sample quality. Analyses were conducted at the laboratory of the Food and Nutrition Research Center of the Institute of Medical Research and Medicinal Plants Studies of Yaoundé, Cameroon. Serum samples were stored frozen in Salapoumbé (which is partly equipped with generators for rural electrification) and transported frozen to Yaoundé in batches for analysis due to the distance between the sites (>700 km). Anthropometric measurements and biological tests were repeated at 3 and 6 months. In addition, the children had systematic deworming with a mebendazole 500 mg tablet every 3 months.

In addition to their habitual diet, all enrolled children were fed two 50 g servings/day (morning and evening) of the assigned IC by their mothers, who had been previously educated on hygienic measures of IC preparation and administration. The fortified IC was prepared with previously boiled warm water. The daily ration of 100 g of IC provided 7.5 mg of iron as ferrous fumarate to children in the iron‐fortified group; otherwise, the two cereals were identical in terms of appearance, taste, and dietary composition (Table 1). The parents and guardians of the children were provided with IC bags every 15 days on a precise and known date. To guarantee the consumption of the planned quantities of IC for each selected child, each household in the study received additional bags of the same IC intended for all of the enrolled child's siblings. At each date of IC supply, parents were questioned for possible adverse events related to the IC administration.

**TABLE 1:** Nutrient content per 100 g of wheat‐based infant cereal (IC) and corresponding recommended micronutrient intakes

	Iron‐fortified IC	Control IC	RNI (1–3 years)^a^	RNI (4–6 years)^a^
Energy	420 kcal	420 kcal		
Fat	10 g	10 g		
Protein	14.5 g	14.5 g		
Carbohydrate	68 g	68 g		
Dietary fiber	2.8 g	2.8 g	ND	ND
Sodium	135 mg	135 mg	<2 g/day	<2 g/day
Calcium	450 mg	450 mg	500 mg/day	600 mg/day
Iron	7.5 mg	0	5.8 mg/day^b^	6.3 mg/day^b^
Zinc	5 mg	5 mg	4.1 mg/day^b^	4.8 mg/day^b^
Vitamin A	1,300 IU	1,300 IU	1,333 IU/day	1,500 IU/day
Vitamin D	180 IU	180 IU	200 IU/day	200 IU/day
Vitamin C	50 mg	50 mg	30 mg/day	30 mg/day
Vitamin B_1_ (thiamin)	0.6 mg	0.6 mg	0.5 mg/day	0.6 mg/day

Note

Recommended intakes of energy, fat, protein, and carbohydrate are not presented because they vary by age, sex, and/or activity level.

Abbreviation: ND, not determined.

^a^ Recommended micronutrient intakes (RNI) established by the Food and Agricultural Organization of the United Nations/World Health Organization/United Nations University (Food and Agricultural Organization of the United Nations & World Health Organization, 2001).

^b^ Value assumes moderate bioavailability.

### 2.1 Ethics

The study protocol was approved by CNERSH and administrative authorization was received from the Cameroon Ministry of Public Health. The study protocol followed local laws and the Declaration of Helsinki. Prior to data collection, parents of enrolled children signed written informed consent after a detailed explanation of the objectives, nature, and risks of the study. All enrolled children who presented with any comorbid condition during the study received free treatment from study medical staff (nurses or physicians). All children who were not enrolled due to severe anemia or severe malnutrition were referred to the local hospital for routine care (e.g., iron supplementation, blood transfusion, inpatient treatment). At the end of the study after code break, all children in the control group received additional ferrous sulfate for 3 months in accordance with standard guidelines (World Health Organization, 2016); anemic children from either group were referred for treatment.

### 2.2 Statistical analysis

The primary endpoints of the study were hemoglobin, serum ferritin adjusted to CRP, serum iron, transferrin saturation and prevalence of anemia, iron deficiency, and iron deficiency anemia. Anemia was defined as hemoglobin of <11 g/dl, consistent with the WHO definition of anemia in children under 5 years of age (World Health Organization & Centers for Disease Control and Prevention, 2007). Iron deficiency was defined as serum (unadjusted) ferritin <12 μg/L for a healthy child or <30 μg/L for a child with an infection or inflammation (World Health Organization, 2001; World Health Organization & Centers for Disease Control and Prevention, 2007). Inflammation was defined as CRP >5 mg/L (Engle‐Stone, Nankap, Ndjebayi, Erhardt, & Brown, 2013; World Health Organization & Food and Agricultural Organization of the United Nations, 1992). Iron deficiency anemia was defined as a hemoglobin level of <11 g/dl associated with a serum (unadjusted) ferritin level of <12 μg/L in a healthy subject or <30 μg/L in a person with an infection or inflammation (World Health Organization & Centers for Disease Control and Prevention, 2007). Among children with inflammation, the serum ferritin level was adjusted to CRP using the BRINDA correction as follows: ferritin_adjusted_ = ferritin_unadjusted_ − (CRP regression coefficient) × (CRP_obs_ − CRP_ref_) (Namaste, Aaron, Varadhan, Peerson, & Suchdev, 2017). The concentration of CRP_ref_ used in this study was 3.75 mg/L (Engle‐Stone et al., 2013). Transferrin saturation was calculated using the total iron‐binding capacity (TIBC; transferrin × 25.1) by the following formula: serum iron × 100/TIBC, where TIBC is in units of µmol/L, transferrin in g/L, serum iron in µmol/L, and transferrin saturation in percent (Beilby et al., 1992; Yamanishi, Iyama, Yamaguchi, Kanakura, & Iwatani, 2003). The secondary endpoints were changes in weight, height, and other anthropometric *z*‐scores.

Analyses were conducted using Epi Info software (version 3.5.4, CDC), WHO Anthro (version 3.2.2, WHO), and SPSS (version 20, SPSS Inc., IBM). The WHO Anthro software was used to calculate weight‐for‐height, height‐for‐age, and weight‐for‐age *z*‐scores in comparison with the WHO standard reference population (World Health Organization, 2006). Descriptive variables for the two groups receiving IC are presented by group and compared using standard *t* tests for continuous measures and chi‐square tests for categorical measures. Biomarkers of iron status are presented as unadjusted means and standard deviation. Because these data were non‐normally distributed, comparisons between the groups for these measures were done using a nonparametric rank‐based ANOVA model at baseline and using a nonparametric rank‐based ANCOVA model at 3 and 6 months, including adjustment for the baseline value. Height and weight as well as the *z*‐scores for the anthropometrics were compared between groups at each time point using a standard parametric ANOVA model (baseline) or ANCOVA model (at 3 and 6 months, again with adjustment for baseline values). Differences between the groups for the prevalence of anemia, iron deficiency, and iron deficiency anemia at baseline, 3, and 6 months were assessed using Fisher's exact test. The threshold of significance was 0.05 for all comparisons.

## 3 RESULTS

**FIGURE 1:**
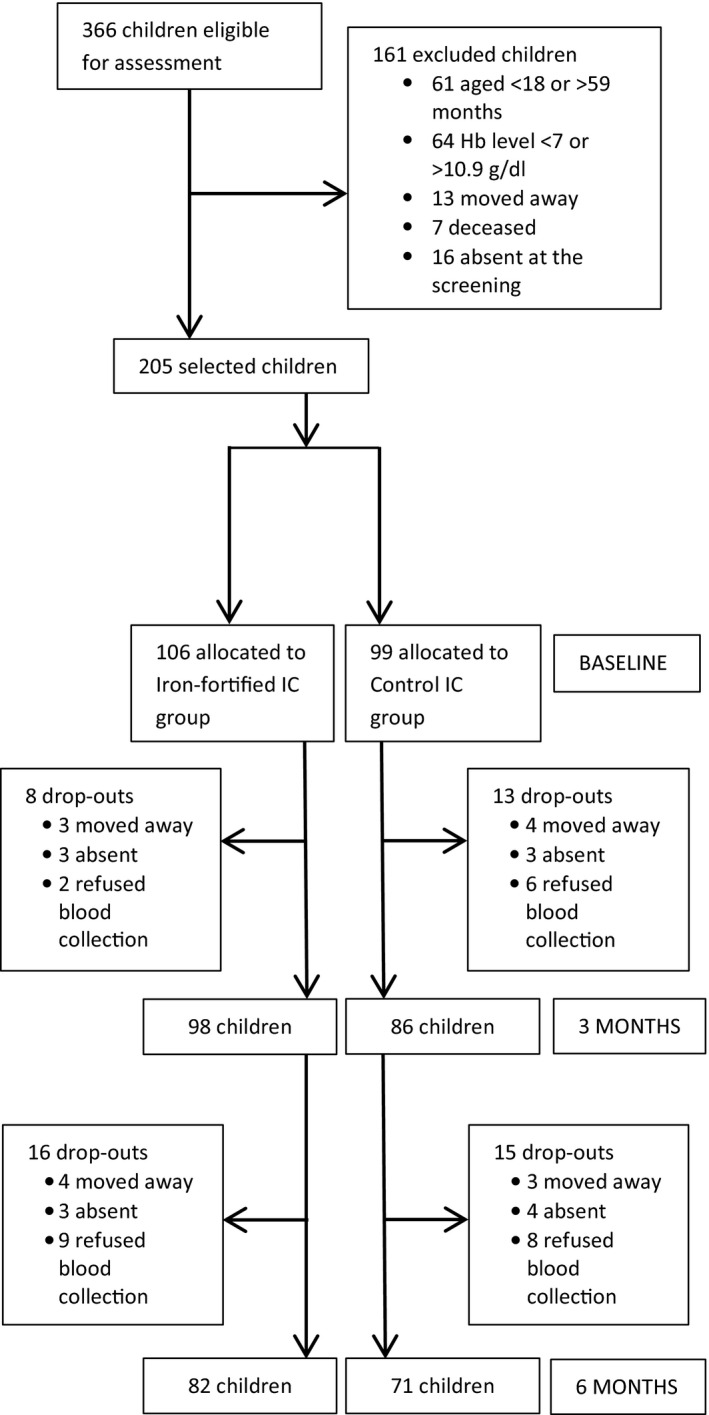
Flow of subject inclusion into trial

Of the 366 children aged 18–59 months identified in the locality of Salapoumbé, 205 children were included the following: 106 children on the south bank who were cluster‐randomized to receive the iron‐fortified IC and 99 children on the north bank who received the control IC. Of the 205 children who participated in the study, 153 children were evaluated at the end of the intervention with a similar dropout rate across the two groups (23% in the iron‐fortified group and 28% in the control group) (Figure 1). The ICs were well tolerated and no side effects were reported in either group. Table 2 presents the demographic characteristics of the enrolled children. Mean age at enrollment was 32.1 months in the iron‐fortified group and 36.1 months in the control group (*p*‐value from *t* test = .009) but no other significant differences were seen between the groups for parental profession or education or family size (Table 2). Overall about 41% of the children had infection or inflammation (CRP > 5 mg/L) at baseline.

**TABLE 2:** Baseline characteristics of the two groups receiving infant cereal (iron‐fortified and control)

	Iron‐fortified IC group (*N* = 106)	Control IC group (*N* = 99)	*p*‐value for difference
Age (months)	32.1 ± 10.9	36.1 ± 10.8	.01
Sex			
Boys, *n* (%)	59 (55.7)	49 (49.5)	.38
Girls, *n* (%)	47 (44.3)	50 (50.5)	
CRP > 5 mg/L, *n* (%)	40 (37.7)	43 (43.4)	.41
Mother's profession			
Civil servant	4 (3.7)	3 (3.0)	.92
Farmer	78 (73.6)	76 (76.2)	
Trader	2 (1.9)	1 (1)	
Homemaker	22 (20.8)	19 (19.8)	
Father's profession			
Civil servant	2 (1.88)	2 (2.0)	.91
Farmer	99 (93.4)	94 (95.0)	
Trader	4 (3.8)	2 (2)	
Fisherman	1 (1)	1 (1)	
Mother's education			
None	70 (66.0)	67 (67.7)	.92
Primary	33 (31.0)	30 (30.3)	
Secondary	3 (3.0)	2 (2.0)	
Father's education			
None	65 (61.3)	60 (60.5)	.92
Primary	26 (24.5)	23 (23.3)	
Secondary	15 (14.2)	16 (16.2)	
Children aged <5 years/household			
1	35 (33.0)	30 (30.0)	.34
2 or 3	70 (66.0)	65 (65.0)	
˃3	1 (1.0)	4 (5.0)	

Note

Values are mean ± standard deviation or count (percentages). *p*‐value for difference computed using standard *t* test for continuous measures and chi‐square test for categorical measures.

Abbreviation: CRP, C‐reactive protein.

The iron, anemia and nutritional status of the enrolled children at baseline, 3 months, and 6 months are presented in Table 3. At baseline, children in the iron‐fortified group had significantly lower hemoglobin levels (*p*‐value from nonparametric ANOVA = .044) and a significantly lower average serum iron level than the control group (*p* = .012). However, the two groups had comparable baseline levels of mean serum ferritin, CRP, and transferrin, mean transferrin saturation, and frequencies of anemia, iron deficiency, and iron deficiency anemia. Mean hemoglobin concentration significantly increased over time in the iron‐fortified group (*p* < .001) whereas change over time in the control group was not significant. Compared to the control group, children consuming the iron‐fortified IC had significantly higher adjusted mean hemoglobin level (10.0 ± 1.8 vs. 9.7 ± 1.4 g/dl, respectively; *p*‐value from nonparametric ANCOVA = .023), ferritin adjusted for CRP (16.1 ± 8.3 vs. 9.5 ± 7.5 μg/L, *p* < .001), serum iron (14.5 ± 3.9 vs. 11.2 ± 4.4 μg/dl; *p* < .001), and transferrin saturation (19.0 ± 17.4 vs. 10.7 ± 12.5%; *p* ˂ .001) at 6 months. Significant group differences for each of these biochemical measures (except ferritin adjusted for CRP) were also observed at 3 months. The prevalence of children with elevated CRP (>5 mg/L) decreased from 43% to 37% in the control group and from 38% to 15% in the iron‐fortified group.

**TABLE 3:** Iron and nutritional status at baseline, 3, and 6 months, by group

	Baseline	*p*‐value for group difference (ANOVA)	3 months	*p*‐value for group difference (ANCOVA)	6 months	*p*‐value for group difference (ANCOVA)	*p*‐value for difference over time (paired *t* test)
Hemoglobin (g/L)							
Fortified IC	9.11 ± 1.20	.044	9.60 ± 1.52	.008	10.00 ± 1.80	.023	<.001
Control IC	9.43 ± 1.01		9.05 ± 1.40		9.66 ± 1.40		.318
Ferritin (µg/L)							
Fortified IC	16.57 ± 11.38	.866	22.14 ± 13.32	.591	19.28 ± 6.76	.007	.1
Control IC	15.61 ± 10.61		21.91 ± 14.42		16.28 ± 6.21		.478
Ferritin adjusted for C‐reactive protein (µg/L)							
Fortified IC	10.17 ± 6.32	.913	15.58 ± 10.35	.817	16.10 ± 8.29	<.001	<.001
Control IC	10.47 ± 6.81		16.44 ± 13.21		9.46 ± 7.51		.614
C‐reactive protein (mg/L)							
Fortified IC	6.16 ± 4.88	.775	3.90 ± 3.10	.720	4.27 ± 3.71	.009	.005
Control IC	6.03 ± 4.44		3.73 ± 2.81		4.74 ± 2.42		.02
Serum iron (µg/dl)							
Fortified IC	10.40 ± 4.25	.012	13.43 ± 3.37	<.001	14.50 ± 3.90	<.001	<.001
Control IC	11.17 ± 2.75		12.36 ± 6.17		11.21 ± 4.42		.982
Transferrin (mg/dl)							
Fortified IC	8.50 ± 7.46	.851	4.95 ± 4.30	<.001	4.72 ± 3.45	<.001	<.001
Control IC	7.30 ± 3.93		7.05 ± 4.87		8.27 ± 5.25		.217
Transferrin saturation (%)							
Fortified IC	9.32 ± 9.33	.317	16.10 ± 10.50	<.001	19.00 ± 17.44	<.001	<.001
Control IC	9.10 ± 8.54		11.80 ± 10.64		10.70 ± 12.50		.667
Weight (kg)							
Fortified IC	11.84 ± 2.10	.20	12.76 ± 2.10	.38	13.41 ± 2.06	.052	<.001
Control IC	12.26 ± 2.35		13.05 ± 2.40		13.36 ± 2.25		<.001
Height (cm)							
Fortified IC	86.22 ± 7.63	.62	88.10 ± 7.43	.35	90.00 ± 7.16	.96	<.001
Control IC	85.65 ± 7.80		88.01 ± 7.72		88.49 ± 7.20		<.001
Weight‐for‐age *z*‐score							
Fortified IC	−1.04 ± 1.33	.69	−0.70 ± 1.29	.18	−0.59 ± 1.17	.016	ND
Control IC	−1.11 ± 1.17		−1.01 ± 1.09		−1.03 ± 0.94		ND
Height‐for‐age *z*‐score							
Fortified IC	−1.72 ± 1.95	.003	−1.67 ± 1.74	.90	−1.62 ± 1.68	.42	ND
Control IC	−2.45 ± 1.53		−2.38 ± 1.51		−2.60 ± 1.31		ND
Weight‐for‐height *z*‐score							
Fortified IC	−0.10 ± 1.17	.001	0.37 ± 1.19	.69	0.51 ± 0.85	.71	ND
Control IC	0.43 ± 1.10		0.56 ± 0.93		0.73 ± 0.77		ND

Note

Values are unadjusted mean ± standard deviation. *p*‐values for difference between fortified IC and control IC groups were calculated using nonparametric ANOVA or ANCOVA model for biochemical measures and standard ANOVA or ANCOVA for anthropometric measures. ANCOVA models include the baseline value as a covariate. *p*‐values for difference between baseline and 6‐months value were calculated using a paired *t* test.

Abbreviation: ND, not determined.

**FIGURE 2:**
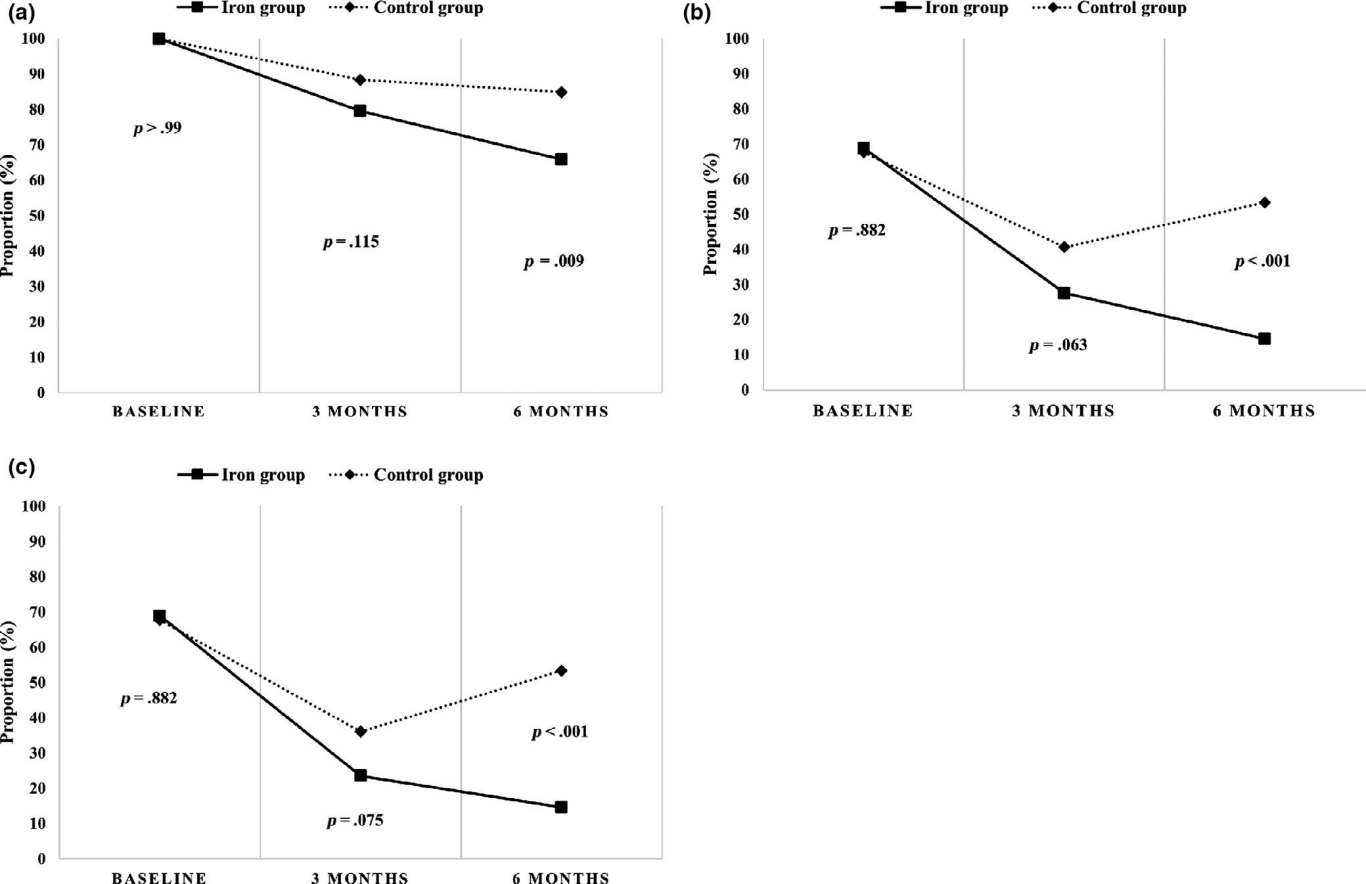
Prevalence of anemia (panel a), iron deficiency (panel b), and iron deficiency anemia (panel c) in both groups at baseline, 3, and 6 months. Comparisons between groups by Fisher's exact test

Figure 2 demonstrates that the proportion of children with anemia, iron deficiency, and iron deficiency anemia significantly decreased from baseline to the end of the intervention in the iron‐fortified group compared to the control group. During this period, the prevalence of anemia decreased by 34% in the iron‐fortified group and by only 15% in the control group (*p*‐value from Fisher's exact test = .009) (panel A). Iron deficiency decreased by 79% in the iron‐fortified group and 21% in the control group (*p*˂.001) (panel B). The prevalence of iron deficiency anemia at baseline, 3, and 6 months was 68.9%, 23.5%, and 14.6%, respectively, in the iron‐fortified group and 67.7%, 36.1%, and 53.4% in the control group (*p* < .001) (panel C).

At baseline, both groups were comparable in weight, height, and weight‐for‐age *z*‐scores, while the iron‐fortified group had higher height‐for‐age and lower weight‐for‐height *z*‐scores than the control group (Table 3). The study population as a whole was mildly to moderately undernourished at baseline, with approximately 56% of children stunted (height‐for‐age *z*‐score <−2) and 25% underweight (weight‐for‐age *z*‐score <−2); only 3% of the children were wasted (weight‐for‐height *z*‐score <−2). At the end of the intervention, the iron‐fortified group had a very small (~50 g) but borderline significant higher weight gain (*p*‐value from ANCOVA = .052) and a significantly higher weight‐for‐age *z*‐score (*p* = .016) compared to the control group (Table 3). Height, height‐for‐age, and weight‐for‐height *z*‐scores were similar in both groups at the end of the intervention. The overall proportion of stunted and underweight children decreased to 52% and 11%, respectively, at the end of the intervention, with no children considered to be wasted; however, anthropometry measures were missing for 27% of the population.

## 4 DISCUSSION

This study found that in addition to their usual diet, daily consumption of wheat‐based IC fortified with 7.5 mg iron as ferrous fumarate among children under 5 years of age improved hemoglobin levels and indicators of iron status and decreased the prevalence of anemia, iron deficiency, and iron deficiency anemia as compared to the control group consuming the same IC without iron. Hemoglobin levels of children in the iron‐fortified group significantly increased by 0.89 ± 1.87 g/dl compared to an increase of 0.23 ± 1.74 g/dl in the control group (note that children in the iron‐fortified group had lower hemoglobin levels than the control group at baseline). These results are comparable to the increase in hemoglobin and reduction in iron deficiency anemia observed in a Kenyan study of children aged 3–8 years who received high‐dose (56 mg/kg) of NaFeEDTA‐enriched maize flour five times per week (Andang'o et al., 2007) and in an Indian study of children aged 6–15 years who received a daily portion of wheat flour fortified with 6 mg of the same iron compound (Muthayya et al., 2012). In addition, a recent study in children aged 6–12 months in India using a similar but rice‐based IC (providing 3.75 mg iron/day as ferrous fumarate) fed for 6 months showed better iron status and reduced risk for anemia and iron deficiency in association with more favorable neurodevelopmental scores (Awasthi et al., 2020). However, our results showed greater efficacy than those reported in another study in India in which children aged 6–13 years received a daily portion of rice fortified with 20 mg iron as micronized ground ferric pyrophosphate (Moretti et al., 2006) and a study in children aged 6–14 years in Côte d'Ivoire who received biscuits fortified with 20 mg electrolytic iron four times per week (Rohner et al., 2010). This difference could be explained by the fact that the fortified IC in the present study also provided vitamin C, known to promote gut iron absorption (de Almeida et al., 2003), and vitamin A, the deficiency of which decreases the absorption of iron (Hurrell & Egli, 2010). Differences in results may also be due in part to the varied iron fortificant compounds used in the studies, which have different bioavailabilities (Hurrell & Egli, 2007), as well as variability in the population characteristics including age, baseline iron status, and inflammation.

In addition, in the present study, significant increases in mean serum iron, CRP‐adjusted ferritin, and transferrin saturation were observed in the iron‐fortified group, and at the end of the intervention, there were significant decreases in the prevalence of anemia, iron deficiency, and iron deficiency anemia in the iron‐fortified group. These results are comparable to those described in the study done in India with NaFeEDTA‐enriched wheat flour (Muthayya et al., 2012). In contrast, the results differ from those in long‐term studies conducted in preschoolers (age 9–71 months) and older children (age 6–11 years) in Sri Lanka (Nestel et al., 2004) and in children under age 6 years in Brazil (Assuncao et al., 2007) that demonstrated no effect of iron‐fortified wheat flour on hemoglobin or anemia. While low hemoglobin status may be due to other factors besides low iron intake and thus an inadequate biomarker to test the efficacy of iron fortification, this difference could also be explained by the fact that the population of the present study consisted exclusively of anemic children, whereas the proportion of anemic children was 8% and 30%, respectively, in the other two clinical trials. Furthermore, the results may differ because the fortificant used in the previously mentioned trials was electrolytic iron, known to have a lower bioavailability than ferrous fumarate, which was used in the present study (Hurrell et al., 2010; Walter, Pizarro, Boy, & Abrams, 2004). The nonsignificant improvement in hemoglobin levels and indicators of iron status (ferritin, serum iron, and transferrin) and the equally nonsignificant decrease in the prevalence of anemia, iron deficiency, and iron deficiency anemia in the control group is unlikely to be due to the cluster randomization, and such a finding has been described in other studies in India (Muthayya et al., 2012) and Côte d'Ivoire (Rohner et al., 2010). These changes in the control group may be related to the vitamin C and vitamin A content of the control IC or to systematic deworming, known for its beneficial effect on reducing digestive parasites and improving absorption of iron from the usual diet (Dreyfuss et al., 2000; Stoltzfus, Dreyfuss, Chwaya, & Albonico, 1997). The increase of iron deficiency and iron deficiency anemia prevalence between midpoint and endpoint in the control group could be explained by the lack of iron supplementation in children recovering from mild malnutrition during the first 3 months of intervention (Macdougall, Moodley, Eyberg, & Quirk, 1982; Velasquez Rodriguez et al., 2007).

There was also indication that nutritional status improved after 6 months of intervention with a small but borderline significant higher weight gain (*p* = .052) and a significantly higher increase in weight‐for‐age *z*‐score (*p* = .016) in the iron‐fortified group compared to the control group. Mean weight‐for‐age *z*‐score at 6 months remained below but moved closer to the WHO median in the fortified group compared to controls. These findings are confirmatory for the beneficial effect of iron fortification of wheat‐based IC on the nutritional status of children, as demonstrated in other studies (Aukett, Parks, Scott, & Wharton, 1986; Barth‐Jaeggi et al., 2015; Briend, Hoque, & Aziz, 1990). This beneficial effect may be due to iron stimulation of appetite in children (Lawless, Latham, Stephenson, Kinoti, & Pertet, 1994; Sachdev, Gera, & Nestel, 2005; Stoltzfus et al., 2004). In addition, the proportion of underweight children in the overall study population decreased from 25% to 11%, likely due to the energy content of the ICs. The high proportion of stunting observed in our population has been observed previously (Pondy, 2016) and is likely due to both poor growth resulting from low dietary diversity and the infrequent inclusion of meats and eggs as complementary foods, as well as overestimation based on the use of WHO growth charts, which are likely nonrepresentative standards for ethnic groups who reside in this area of Cameroon and who generally have a shorter stature (Funk et al., 2019). Growth charts specific for this population are not yet available.

Although the amount of iron provided by the fortified cereal was relatively small, our group has previously reported a pattern of complementary feeding in Cameroon that consists mostly of traditional and indigenous foods such as corn gruel, potato puree, banana, and other starch‐based foods that lack diversity and do not meet the dietary iron needs of growing children (Kana‐Sop et al., 2013). While fish is also commonly consumed, the amount eaten by children is generally insufficient to cover their iron needs (Kana‐Sop et al., 2013). The median intake of fortified wheat flour by children aged 12–59 months in Cameroon has been reported to be 31 g/day, representing an intake of 1.86 mg/day of iron from this food source (Engle‐Stone, Nankap, et al., 2017). Therefore, the iron provided by the fortified IC (7.5 mg/day) likely represented a sizable proportion of total daily intake.

An important limitation of this study is that only two clusters were randomized, and thus, the differences observed at the end of the study between the clusters located north and south of the river could theoretically be due to differences in living conditions in the two areas; however, as the two areas are largely comparable in terms of customs, diet, level of literacy, and agro‐pastoral activities, this seems to be an unlikely explanation of the results observed. Moreover, the lack of assessment of malaria in both groups during the intervention did not permit us to conclude on the safety of iron fortification in this endemic malaria area as shown in other studies (Prentice, Verhoef, & Cerami, 2013; Zlotkin et al., 2013). However, the iron concentration of the IC (3.75 mg/serving) is much lower than that of micronutrient powders (12.5 mg/serving) previously associated with increased risk of hospitalization and mortality in malaria‐endemic areas (Sazawal et al., 2006). This lower iron dose within a food matrix is unlikely to promote the appearance of plasma nontransferrin bound iron that can enhance bacterial virulence (Brittenham, 2012). The lack of information on compliance or the amount of cereal consumed is an additional study limitation. Finally, no formal adjustment for multiplicity was performed to account for multiple primary endpoints (seven parameters). However, if a Bonferroni correction is applied (*p*‐value of interest = .05/7 or .00714), differences between groups for six of the seven endpoints remain statistically significant at study end, with only hemoglobin no longer significant. Adjusting for multiplicity does not change the overall interpretation of the study results.

The strengths of the study include the rural setting and subject selection process, which resulted in a study population that is generally representative of this area of Cameroon and other regions of Africa. In addition, the cluster‐randomized design helped minimize treatment “contamination” between intervention and control participants, which can reduce the apparent effectiveness of an intervention (Torgerson, 2001).

## 5 CONCLUSION

In conclusion, these results suggest that daily consumption of micronutrient‐fortified IC enriched with 7.5 mg of iron as ferrous fumarate for 6 months improved hemoglobin and iron status and decreased the prevalence of anemia, iron deficiency, and iron deficiency anemia in children under 5 years of age in a malaria‐endemic setting, in which it is more difficult to demonstrate a positive effect due to the high infection rate. In addition, the children receiving the iron‐fortified IC had improved growth parameters. Given that most households in this region consume wheat flour, a geographically expanded program of iron fortification of affordable cereals in association with other public health interventions, such as prevention of malaria and promotion of immunization, could help to prevent and correct iron deficiency anemia and improve nutritional status in the locality of Salapoumbé, and possibly throughout Cameroon and beyond.

## CONFLICT OF INTEREST

NPH is employed by Société des Produits Nestlé SA. No other author has any conflict of interest to report.

## ETHICAL APPROVAL

This study was approved by the Comité National d'Ethique et de la Recherche pour la Santé Humaine du Cameroun, and administrative authorization was received from the Cameroon Ministry of Public Health.

## INFORMED CONSENT

Written informed consent was obtained from the parents of all study participants.

## References

[fsn31639-bib-0001] Andang'o, P. E. , Osendarp, S. J. , Ayah, R. , West, C. E. , Mwaniki, D. L. , De Wolf, C. A. , … Verhoef, H. (2007). Efficacy of iron‐fortified whole maize flour on iron status of schoolchildren in Kenya: A randomised controlled trial. Lancet, 369(9575), 1799–1806. 10.1016/S0140-6736(07)60817-4 17531887

[fsn31639-bib-0002] Assuncao, M. C. , Santos, I. S. , Barros, A. J. , Gigante, D. P. , & Victora, C. G. (2007). Effect of iron fortification of flour on anemia in preschool children in Pelotas, Brazil. Revista de Saude Publica, 41(4), 539–548. 17589751 10.1590/s0034-89102006005000031

[fsn31639-bib-0003] Aukett, M. A. , Parks, Y. A. , Scott, P. H. , & Wharton, B. A. (1986). Treatment with iron increases weight gain and psychomotor development. Archives of Disease in Childhood, 61(9), 849–857. 10.1136/adc.61.9.849 2429622 PMC1778027

[fsn31639-bib-0004] Awasthi, S. , Reddy, N. U. , Mitra, M. , Singh, S. , Ganguly, S. , Jankovic, I. , … Ghosh, A. (2020). Micronutrient‐fortified infant cereal improves hemoglobin status and reduces iron deficiency anemia in Indian infants: An effectiveness study. British Journal of Nutrition, 123, 782–791. 10.1017/S0007114519003386 PMC7054248 31896356

[fsn31639-bib-0005] Baltussen, R. , Knai, C. , & Sharan, M. (2004). Iron fortification and iron supplementation are cost‐effective interventions to reduce iron deficiency in four subregions of the world. Journal of Nutrition, 134(10), 2678–2684. 10.1093/jn/134.10.2678 15465766

[fsn31639-bib-0006] Barth‐Jaeggi, T. , Moretti, D. , Kvalsvig, J. , Holding, P. A. , Njenga, J. , Mwangi, A. , … Zimmermann, M. B. (2015). In‐home fortification with 2.5 mg iron as NaFeEDTA does not reduce anaemia but increases weight gain: A randomised controlled trial in Kenyan infants. Maternal & Child Nutrition, 11(Suppl 4), 151–162. 10.1111/mcn.12163 25420455 PMC6860217

[fsn31639-bib-0007] Beard, J. L. (2008). Why iron deficiency is important in infant development. Journal of Nutrition, 138(12), 2534–2536. 10.1093/jn/138.12.2534 19022985 PMC3415871

[fsn31639-bib-0008] Beilby, J. , Olynyk, J. , Ching, S. , Prins, A. , Swanson, N. , Reed, W. , … Garcia‐Webb, P. (1992). Transferrin index: An alternative method for calculating the iron saturation of transferrin. Clinical Chemistry, 38(10), 2078–2081. 10.1093/clinchem/38.10.2078 1394993

[fsn31639-bib-0009] Bouhouch, R. R. , El‐Fadeli, S. , Andersson, M. , Aboussad, A. , Chabaa, L. , Zeder, C. , … Zimmermann, M. B. (2016). Effects of wheat‐flour biscuits fortified with iron and EDTA, alone and in combination, on blood lead concentration, iron status, and cognition in children: A double‐blind randomized controlled trial. American Journal of Clinical Nutrition, 104(5), 1318–1326. 10.3945/ajcn.115.129346 27733396

[fsn31639-bib-0010] Briend, A. , Hoque, B. A. , & Aziz, K. M. (1990). Iron in tubewell water and linear growth in rural Bangladesh. Archives of Disease in Childhood, 65(2), 224–225. 10.1136/adc.65.2.224 2317069 PMC1792232

[fsn31639-bib-0011] Brittenham, G. M. (2012). Safety of iron fortification and supplementation in malaria‐endemic areas. Nestlé Nutrition Institute Workshop Series, 70, 117–127. 10.1159/000337674 25762976 PMC4353606

[fsn31639-bib-0012] Dallman, P. R. , Siimes, M. A. , & Stekel, A. (1980). Iron deficiency in infancy and childhood. American Journal of Clinical Nutrition, 33(1), 86–118. 10.1093/ajcn/33.1.86 6986756

[fsn31639-bib-0013] Das, J. K. , Salam, R. A. , Kumar, R. , & Bhutta, Z. A. (2013). Micronutrient fortification of food and its impact on woman and child health: A systematic review. Systematic Reviews, 2, 67 10.1186/2046-4053-2-67 23971426 PMC3765883

[fsn31639-bib-0014] de Almeida, C. A. N. , Crott, G. C. I. , Ricco, R. G. , Del Ciampo, L. A. , Dutra‐de‐Oliveira, J. E. , & Cantolini, A. (2003). Control of iron‐deficiency anaemia in Brazilian preschool children using iron‐fortified orange juice. Nutrition Research, 23(1), 27–33. 10.1016/S0271-5317(02)00487-6

[fsn31639-bib-0015] Dreyfuss, M. L. , Stoltzfus, R. J. , Shrestha, J. B. , Pradhan, E. K. , LeClerq, S. C. , Khatry, S. K. , … West, K. P. Jr (2000). Hookworms, malaria and vitamin A deficiency contribute to anemia and iron deficiency among pregnant women in the plains of Nepal. Journal of Nutrition, 130(10), 2527–2536. 10.1093/jn/130.10.2527 11015485

[fsn31639-bib-0016] Eichler, K. , Wieser, S. , Ruthemann, I. , & Brugger, U. (2012). Effects of micronutrient fortified milk and cereal food for infants and children: A systematic review. BMC Public Health, 12, 506 10.1186/1471-2458-12-506 22770558 PMC3444335

[fsn31639-bib-0017] Engle‐Stone, R. , Aaron, G. J. , Huang, J. , Wirth, J. P. , Namaste, S. M. , Williams, A. M. , … Suchdev, P. S. (2017). Predictors of anemia in preschool children: Biomarkers Reflecting Inflammation and Nutritional Determinants of Anemia (BRINDA) project. American Journal of Clinical Nutrition, 106(Suppl 1), 402S–415S. 10.3945/ajcn.116.142323 28615260 PMC5490650

[fsn31639-bib-0018] Engle‐Stone, R. , Nankap, M. , Ndjebayi, A. O. , Allen, L. H. , Shahab‐Ferdows, S. , Hampel, D. , … Brown, K. H. (2017). Iron, zinc, folate, and vitamin B‐12 status increased among women and children in Yaounde and Douala, Cameroon, 1 year after introducing fortified wheat flour. Journal of Nutrition, 147(7), 1426–1436. 10.3945/jn.116.245076 28592513 PMC5483962

[fsn31639-bib-0019] Engle‐Stone, R. , Nankap, M. , Ndjebayi, A. O. , Erhardt, J. G. , & Brown, K. H. (2013). Plasma ferritin and soluble transferrin receptor concentrations and body iron stores identify similar risk factors for iron deficiency but result in different estimates of the national prevalence of iron deficiency and iron‐deficiency anemia among women and children in Cameroon. Journal of Nutrition, 143(3), 369–377. 10.3945/jn.112.167775 23343673

[fsn31639-bib-0020] Food and Agricultural Organization of the United Nations , & World Health Organization (2001). Human vitamin and mineral requirements: Report of a joint FAO/WHO expert consultation, Bangkok, Thailand. Rome, Italy.

[fsn31639-bib-0021] Food Fortification Initiative (2018). Regional activity. Available from: http://ffinetwork.org/regional_activity/africa.php. [last accessed 27 February 2020].

[fsn31639-bib-0022] Funk, S. M. , Guerra, B. P. , Ickowitz, A. , Poni, N. A. , Abdou, M. A. , Sibama, Y. H. , … Fa, J. E. (2019). WHO child growth standards for Pygmies: One size fits all? Biorxiv, 591172 10.1101/591172

[fsn31639-bib-0023] Gera, T. , Sachdev, H. S. , & Boy, E. (2012). Effect of iron‐fortified foods on hematologic and biological outcomes: Systematic review of randomized controlled trials. American Journal of Clinical Nutrition, 96(2), 309–324. 10.3945/ajcn.111.031500 22760566

[fsn31639-bib-0024] Glinz, D. , Hurrell, R. F. , Ouattara, M. , Zimmermann, M. B. , Brittenham, G. M. , Adiossan, L. G. , … Wegmuller, R. (2015). The effect of iron‐fortified complementary food and intermittent preventive treatment of malaria on anaemia in 12‐ to 36‐month‐old children: A cluster‐randomised controlled trial. Malaria Journal, 14, 347 10.1186/s12936-015-0872-3 26377199 PMC4573684

[fsn31639-bib-0025] Glinz, D. , Wegmuller, R. , Ouattara, M. , Diakite, V. G. , Aaron, G. J. , Hofer, L. , … Hurrell, R. F. (2017). Iron fortified complementary foods containing a mixture of sodium iron EDTA with either ferrous fumarate or ferric pyrophosphate reduce iron deficiency anemia in 12‐ to 36‐month‐old children in a malaria endemic setting: A secondary analysis of a cluster‐randomized controlled trial. Nutrients, 9(7), 759 10.3390/nu9070759 PMC5537873 28708072

[fsn31639-bib-0026] Horton, S. (2006). The economics of food fortification. Journal of Nutrition, 136(4), 1068–1071. 10.1093/jn/136.4.1068 16549479

[fsn31639-bib-0027] Hurrell, R. F. , & Egli, I. (2007). Optimizing the bioavailability of iron compounds for food fortification In Kraemer K., & Zimmermann M. B. (Eds.), Nutritional anemia (pp. 77–90). Basel, Switzerland: Sight and Life Press.

[fsn31639-bib-0028] Hurrell, R. , & Egli, I. (2010). Iron bioavailability and dietary reference values. American Journal of Clinical Nutrition, 91(5), 1461S–1467S. 10.3945/ajcn.2010.28674F 20200263

[fsn31639-bib-0029] Hurrell, R. , Ranum, P. , de Pee, S. , Biebinger, R. , Hulthen, L. , Johnson, Q. , & Lynch, S. (2010). Revised recommendations for iron fortification of wheat flour and an evaluation of the expected impact of current national wheat flour fortification programs. Food and Nutrition Bulletin, 31(1 Suppl), S7–S21. 10.1177/15648265100311S102 20629349

[fsn31639-bib-0030] Institut National de la Statistique (INS) , & ICF International (2012). Enquête Démographique et de Santé et à Indicateurs Multiples du Cameroun 2011. Available from: https://dhsprogram.com/pubs/pdf/fr260/fr260.pdf. [last accessed 27 February 2020].

[fsn31639-bib-0031] Kana‐Sop, M. M. , Gouado, I. , Mananga, M. J. , Ekoule, L. D. , Amvan Zollo, P. H. , & Ekoe, T. (2013). Evaluation of nutritional status of young children aged 0–2 years in the Douala city (Cameroon), survey of some practices during diversification of complementary foods. African Journal of Food Science and Technology, 4(2), 29–34.

[fsn31639-bib-0032] Lawless, J. W. , Latham, M. C. , Stephenson, L. S. , Kinoti, S. N. , & Pertet, A. M. (1994). Iron supplementation improves appetite and growth in anemic Kenyan primary school children. Journal of Nutrition, 124(5), 645–654. 10.1093/jn/124.5.645 8169656

[fsn31639-bib-0033] Loukas, A. , Hotez, P. J. , Diemert, D. , Yazdanbakhsh, M. , McCarthy, J. S. , Correa‐Oliveira, R. , … Bethony, J. M. (2016). Hookworm infection. Nature Reviews Disease Primers, 2, 16088 10.1038/nrdp.2016.88 27929101

[fsn31639-bib-0034] Macdougall, L. G. , Moodley, G. , Eyberg, C. , & Quirk, M. (1982). Mechanisms of anemia in protein‐energy malnutrition in Johannesburg. American Journal of Clinical Nutrition, 35(2), 229–235. 10.1093/ajcn/35.2.229 6461244

[fsn31639-bib-0035] Mananga, M. J. , Kana‐Sop, M. M. , Nolla, N. P. , Tetanye, E. , & Gouado, G. I. (2014). Feeding practices, food and nutrition insecurity of infants and their mothers in Bangang rural community, Cameroon. Journal of Nutrition & Food Sciences, 4(2). 10.4172/2155-9600.1000264

[fsn31639-bib-0036] McLean, E. , Cogswell, M. , Egli, I. , Wojdyla, D. , & de Benoist, B. (2009). Worldwide prevalence of anaemia, WHO Vitamin and Mineral Nutrition Information System, 1993–2005. Public Health Nutrition, 12(4), 444–454. 10.1017/S1368980008002401 18498676

[fsn31639-bib-0037] Moretti, D. , Zimmermann, M. B. , Muthayya, S. , Thankachan, P. , Lee, T. C. , Kurpad, A. V. , & Hurrell, R. F. (2006). Extruded rice fortified with micronized ground ferric pyrophosphate reduces iron deficiency in Indian schoolchildren: A double‐blind randomized controlled trial. American Journal of Clinical Nutrition, 84(4), 822–829. 10.1093/ajcn/84.4.822 17023709

[fsn31639-bib-0038] Muthayya, S. , Thankachan, P. , Hirve, S. , Amalrajan, V. , Thomas, T. , Lubree, H. , … Kurpad, A. V. (2012). Iron fortification of whole wheat flour reduces iron deficiency and iron deficiency anemia and increases body iron stores in Indian school‐aged children. Journal of Nutrition, 142(11), 1997–2003. 10.3945/jn.111.155135 23014487

[fsn31639-bib-0039] Namaste, S. M. , Aaron, G. J. , Varadhan, R. , Peerson, J. M. , Suchdev, P. S. , & BRINDA Working Group (2017). Methodologic approach for the Biomarkers Reflecting Inflammation and Nutritional Determinants of Anemia (BRINDA) project. American Journal of Clinical Nutrition, 106(Suppl 1), 333S–347S. 10.3945/ajcn.116.142273 28615254 PMC5490643

[fsn31639-bib-0040] Nestel, P. , Nalubola, R. , Sivakaneshan, R. , Wickramasinghe, A. R. , Atukorala, S. , & Wickramanayake, T. (2004). The use of iron‐fortified wheat flour to reduce anemia among the estate population in Sri Lanka. International Journal for Vitamin and Nutrition Research, 74(1), 35–51. 10.1024/0300-9831.74.1.35 15060899

[fsn31639-bib-0041] Nutrition International (2018). In the World. Available from: https://www.nutritionintl.org/fr/dans‐le‐monde/. [last accessed 27 February 2020].

[fsn31639-bib-0042] Pachon, H. , Spohrer, R. , Mei, Z. , & Serdula, M. K. (2015). Evidence of the effectiveness of flour fortification programs on iron status and anemia: A systematic review. Nutrition Reviews, 73(11), 780–795. 10.1093/nutrit/nuv037 26433017 PMC9052958

[fsn31639-bib-0043] Pondy, D. B. (2016). [Nutritional status and anemia in children aged 6 to 59 months in Salapoumbé, forest area in Cameroon]. Health Sciences and Diseases Available from: https://www.hsd‐fmsb.org/index.php/hsd/thesis/view/462. [last accessed 26 February 2020].

[fsn31639-bib-0044] Prentice, A. M. , Verhoef, H. , & Cerami, C. (2013). Iron fortification and malaria risk in children. JAMA, 310(9), 914–915. 10.1001/jama.2013.6771 24002276 PMC6136145

[fsn31639-bib-0045] Rohner, F. , Zimmermann, M. B. , Amon, R. J. , Vounatsou, P. , Tschannen, A. B. , N'Goran, E. K. , … Hurrell, R. F. (2010). In a randomized controlled trial of iron fortification, anthelmintic treatment, and intermittent preventive treatment of malaria for anemia control in Ivorian children, only anthelmintic treatment shows modest benefit. Journal of Nutrition, 140(3), 635–641. 10.3945/jn.109.114256 20107144

[fsn31639-bib-0046] Sachdev, H. , Gera, T. , & Nestel, P. (2005). Effect of iron supplementation on mental and motor development in children: Systematic review of randomised controlled trials. Public Health Nutrition, 8(2), 117–132. 10.1079/PHN2004677 15877905

[fsn31639-bib-0047] Sazawal, S. , Black, R. E. , Ramsan, M. , Chwaya, H. M. , Stoltzfus, R. J. , Dutta, A. , … Kabole, F. M. (2006). Effects of routine prophylactic supplementation with iron and folic acid on admission to hospital and mortality in preschool children in a high malaria transmission setting: Community‐based, randomised, placebo‐controlled trial. Lancet, 367(9505), 133–143. 10.1016/S0140-6736(06)67962-2 16413877

[fsn31639-bib-0048] Stoltzfus, R. J. , Chway, H. M. , Montresor, A. , Tielsch, J. M. , Jape, J. K. , Albonico, M. , & Savioli, L. (2004). Low dose daily iron supplementation improves iron status and appetite but not anemia, whereas quarterly anthelminthic treatment improves growth, appetite and anemia in Zanzibari preschool children. Journal of Nutrition, 134(2), 348–356. 10.1093/jn/134.2.348 14747671

[fsn31639-bib-0049] Stoltzfus, R. J. , Dreyfuss, M. L. , Chwaya, H. M. , & Albonico, M. (1997). Hookworm control as a strategy to prevent iron deficiency. Nutrition Reviews, 55(6), 223–232. 10.1111/j.1753-4887.1997.tb01609.x 9279058

[fsn31639-bib-0050] Sun, J. , Huang, J. , Li, W. , Wang, L. , Wang, A. , Huo, J. , … Chen, C. (2007). Effects of wheat flour fortified with different iron fortificants on iron status and anemia prevalence in iron deficient anemic students in Northern China. Asia Pacific Journal of Clinical Nutrition, 16(1), 116–121. 17215188

[fsn31639-bib-0051] Torgerson, D. J. (2001). Contamination in trials: Is cluster randomisation the answer? BMJ, 322(7282), 355–357. 11159665 10.1136/bmj.322.7282.355 PMC1119583

[fsn31639-bib-0052] Velasquez Rodriguez, C. M. , Parra Sosa, B. , Morales Mira, G. , Agudelo Ochoa, G. , Cardona Henao, O. , Bernal Parra, C. , … Betancur Acosta, M. (2007). “Free” iron, transferrin and ferritin levels in serum and their relation with severe malnutrition. Anales de Pediatría, 66(1), 17–23. 17266850 10.1157/13097353

[fsn31639-bib-0053] Walter, T. , Pizarro, F. , Boy, E. , & Abrams, S. A. (2004). The poor bioavailability of elemental iron in corn masa flour is not affected by disodium EDTA. Journal of Nutrition, 134(2), 380–383. 10.1093/jn/134.2.380 14747675

[fsn31639-bib-0054] World Health Organization (2001). Iron deficiency anaemia assessment, prevention and control: A guide for program managers. Geneva, Switzerland: WHO.

[fsn31639-bib-0055] World Health Organization (2006). WHO child growth standards: Length/height‐for‐age, weight‐for‐age, weight‐for‐length, weight‐for‐height and body mass index‐for‐age: Methods and development. Geneva, Switzerland: WHO.

[fsn31639-bib-0056] World Health Organization (2016). Guideline: Daily iron supplementation in infants and children. Geneva, Switzerland: WHO. 27195348

[fsn31639-bib-0057] World Health Organization , & Centers for Disease Control and Prevention (2007). Assessing the iron status of populations. Second edition, including literature reviews. Geneva, Switzerland: WHO.

[fsn31639-bib-0058] World Health Organization , & Food and Agricultural Organization of the United Nations (1992). International conference on nutrition: World declaration and plan of action for nutrition. Rome, Italy.

[fsn31639-bib-0059] Yamanishi, H. , Iyama, S. , Yamaguchi, Y. , Kanakura, Y. , & Iwatani, Y. (2003). Total iron‐binding capacity calculated from serum transferrin concentration or serum iron concentration and unsaturated iron‐binding capacity. Clinical Chemistry, 49(1), 175–178. 10.1373/49.1.175 12507977

[fsn31639-bib-0060] Zlotkin, S. , Newton, S. , Aimone, A. M. , Azindow, I. , Amenga‐Etego, S. , Tchum, K. , … Owusu‐Agyei, S. (2013). Effect of iron fortification on malaria incidence in infants and young children in Ghana: A randomized trial. JAMA, 310(9), 938–947. 10.1001/jama.2013.277129 24002280

